# Genetic Mapping of Quantitative Trait Loci for Yield-Affecting Traits in a Barley Doubled Haploid Population Derived from Clipper × Sahara 3771

**DOI:** 10.3389/fpls.2017.00688

**Published:** 2017-07-18

**Authors:** Ulduz Vafadarshamasbi, Seyed Hossein Jamali, Behzad Sadeghzadeh, Babak Abdollahi Mandoulakani

**Affiliations:** ^1^Department of Agricultural Biotechnology, Maragheh Branch, Islamic Azad University Maragheh, Iran; ^2^Seed and Plant Certification and Registration Institute, Agricultural Research, Education and Extension Organization Karaj, Iran; ^3^Dryland Agricultural Research Institute, Agricultural Research, Education and Extension Organization Maragheh, Iran; ^4^Department of Plant Breeding and Biotechnology, Faculty of Agriculture, Urmia University Urmia, Iran

**Keywords:** barley (*Hordeum vulgare* L.), grain yield, linkage analysis, morphological traits, quantitative trait loci (QTL)

## Abstract

Many traits play essential roles in determining crop yield. Wide variation for morphological traits exists in *Hordeum vulgare L*., but the genetic basis of this morphological variation is largely unknown. To understand genetic basis controlling morphological traits affecting yield, a barley doubled haploid population (146 individuals) derived from Clipper × Sahara 3771 was used to map chromosome regions underlying days to awn appearance, plant height, fertile spike number, flag leaf length, spike length, harvest index, seed number per plant, thousands kernel weight, and grain yield. Twenty-seven QTLs for nine traits were mapped to the barley genome that described 3–69% of phenotypic variations; and some genomic regions harbor a given QTL for more than one trait. Out of 27 QTLs identified, 19 QTLs were novel. Chromosomal regions on 1H, 2H, 4H, and 6H associated with seed grain yield, and chromosome regions on 2H and 6H had major effects on grain yield (GY). One major QTL for seed number per plant was flanked by marker VRS1-KSUF15 on chromosome 2H. This QTL was also associated with GY. Some loci controlling thousands kernel weight (TKW), fertile spike number (FSN), and GY were the same. The major grain yield QTL detected on linkage PSR167 co-localized with TAM10. Two major QTLs controlling TKW and FSN were also mapped at this locus. Eight QTLs on chromosomes 1H, 2H, 3H, 4H, 5H, 6H, and 7H consistently affected spike characteristics. One major QTL (ANIONT1A-TACMD) on 4H affected both spike length (SL) and spike number explained 9 and 5% of the variation of SL and FSN, respectively. In conclusion, this study could cast some light on the genetic basis of the studied pivotal traits. Moreover, fine mapping of the identified major effect markers may facilitate the application of molecular markers in barley breeding programs.

## Introduction

Barley (*Hordeum vulgare* L.) is the fourth among cereal crops in grain production throughout the world (Asare et al., 2011; FAOSTAT, 2017). Barley is highly variable in utilization (i.e., animal feed, alcohol production, people food) and adaptation to marginal and subsistence environments. The principal focus of most commercial barley breeding programs has been on improving yield by developing new cultivars with high grain yield (GY) and high quality characteristics for different soils and climates (Arpaili and Yagmur, 2015).

In the process of crop improvement, determining the genetic basis of agronomic traits has been a scientific principle (Pasam et al., 2012). Most of the agronomical traits are quantitative and a number of genetic loci usually controls these traits (Ren et al., 2005). Quantitative trait loci (QTLs) mapping technology examine genetically many complex traits and QTL (Liu et al., 2015). This technology maps these loci with considering their positions in the genome and compares gene action, phenotypic effects, pleiotropic effects and epistasis interactions with other QTLs (Xiao et al., 1996; Zhu et al., 1999).

In barley, the identification of QTLs underlying main agronomic traits like GY (Thomas et al., 1998; Liu et al., 2015), malting quality (Borem et al., 1999; Beecher et al., 2001), disease resistance (Chen et al., 1994; Steffenson et al., 1996), drought tolerance (Mohammadi et al., 2005; Peighambari et al., 2005), and salt tolerance (Siahsar and Narouei, 2010; Aminfar et al., 2011) has been reported using molecular markers.

Up to now, limited information is available on the inheritance of morphological traits in doubled haploid (DH) population derived from Clipper × Sahara 3771. DH populations are very valuable material for genetic and molecular studies such as inheritance of quantitative traits, QTL mapping, genomics, gene identification, whole genome mapping due to less time requirement to make release of cultivars with desirable traits (Hussain et al., 2012). Genetic studies on the identification of loci controlling morphological variation show the complex genetic control of many quantitatively inherited traits in barley (Gyenis et al., 2007). The identification of QTLs associated with traits affecting yield is a significant starting point for transferring and pyramiding genes that can provide improvement of barley productivity (Sishen et al., 2007; Sadeghzadeh et al., 2015).

Heading date is a vitally important trait in adapting cereal species to drought conditions and maximizing yield potential (Bezant et al., 1996). Minimizing risk of lodging and increasing the harvest index (HI) can be controlled by plant height trait (Bezant et al., 1996). Heading date and plant height are influenced by many QTLs. Such traits usually exhibit complex inheritance involving multiple genes and environmental effect on trait development (Kjaer et al., 1995).

Spike morphology is related to GY because of its effects on grain number. Genetic gains in GY have historically been improved by changes in grain number per spike and spikes per square meter, with little change in individual grain weight. Hence, modifying the spike morphology to increase grain number may provide new opportunities for higher GY potential (Gaju et al., 2009; Zhou, 2015). In barley studies, the importance of flag leaf in determining GY has already been reported (Thorne, 1965; Yap and Harvey, 1972; Tungland et al., 1987; Zheng, 1999). Flag leaf could produce a large proportion of the carbohydrates stored in grains (Li et al., 1998). The morphological traits of flag leaf such as size and shape would be one of the best components in producing high GY (Hirota et al., 1990; Chen et al., 1995).

Ren et al. (2013) mapped the correlation QTL of agronomic and quality traits associated with GY in a DH population of barley. In another study, 16 QTLs associated with 3 morphological traits including flag leaf area, flag leaf length (FLL), and flag leaf width were identified in 2 years, which were located on chromosomes 2H, 3H, 4H, and 7H, respectively (Liu et al., 2015). Thousand kernel weight associated with directly the GY and quality of cereal crops, also with affecting on seedling vigor and growth, can indirectly affect GY (Wiersma et al., 2001; Botwright et al., 2002).

Genetic analyses have demonstrated close relationships between plant height, days to heading, FLL, spike length (SL), kernel weight with barley GY (Pasam et al., 2012); hence, our data may provide information for marker assisted selection (MAS) in barley breeding for high yield. The objective of this study was to determine the chromosomal location and phenotypic effects of QTLs associated with yield-affecting agro-morphological traits, GY and its component traits under controlled conditions. Molecular markers associated with QTLs identified would enhance progress in barley breeding programs for higher yield.

## Materials and Methods

The plant material used for the map construction and phenotyping was a DH population derived from a cross between the Australian cultivar 2-rowed Clipper (high yield) and Algerian 6-rowed Sahara 3771 (low yield landrace). The population was produced by the *Hordeum bulbosum* method (Finnie et al., 1989). The DH population and molecular data were kindly provided by the University of Western Australia. The measured traits were days from sowing to awn appearance (DAA), plant height (PLH), FLL, SL, fertile spike number per plant (FSN), seed number per plant (SN), thousands kernel weight (TKW), HI, and GY.

Twelve pre-germinated seeds from each DH line and their parents were sown under glasshouse conditions in soil surface of each plastic-lined pot. The experiment was carried out based on a randomized complete block design with two replications. Plants were grown in glasshouse at 25°C day/15°C night temperature and 10 h natural sunlight photoperiod. The pots were filled with a poor sandy soil with pH 6. The pots were fertilized with basal nutrients (in mg/kg of dry soil) 90 mono-potassium phosphate (KH_2_PO_4_), 145 potassium sulfate (K_2_SO_4_), 20 magnesium sulfate hepta-hydrate (MgSO_4_.7H_2_O), 150 calcium chloride dihydrate (CaCl_2_.2H_2_O), 2 copper sulfate pentahydrates (CuSO_4_.5H_2_O), 0.7 boric acid (H_3_BO_3_), 15 manganese sulfate monohydrate (MnSO_4_.H_2_O), 0.8 mg Zn/kg soil as zinc sulfate hepta-hydrate (ZnSO_4_.7H_2_O), 0.2 sodium molybdate dihydrate (Na_2_MoO_4_.2H_2_O), and 93 ammonium nitrate (NH_4_NO_3_). To minimize the effect of local microenvironment variation, the pots were rotated within a block daily. Plants were watered with double-deionized water daily by weight in glasshouse, keeping water content at 90% of the field capacity.

A genetic linkage map of ‘Clipper’ × ‘Sahara 3771’ population was developed by Karakousis et al. (2003). We mapped QTLs for the studied traits across the seven barley chromosomes using 335-restriction fragment length polymorphism (RFLP), simple sequence repeats (SSR) and morphological markers from this paper. QTL linkage mapping was performed using the software package QTL Network (Yang et al., 2007). To define individual adjacent QTL, we consider a minimum separation of 10 cM (“filtration window”).

Quantitative trait loci effects were simulated using Bayesian method of mixed linear model via Gibbs sampling (Sadeghzadeh et al., 2008). A *P*-value is obtained for each of the estimates of QTL effects by this analysis. A threshold of *P*-value less than 0.05 was considered as significance for QTL effect estimation. Correlation and QTL analyses were estimated for the data from each block (using Pearson’s coefficient; two-tailed test), all data were analyzed to determine if there was a relationship between all the measured traits in the DH lines using SPSS software (Version 10; SPSS Inc., Chicago, IL, United States). Heritability was calculated for each trait using ANOVA analysis (using Version 10; SPSS Inc., Chicago, IL, United States software).

## Results

**FIGURE 1:**
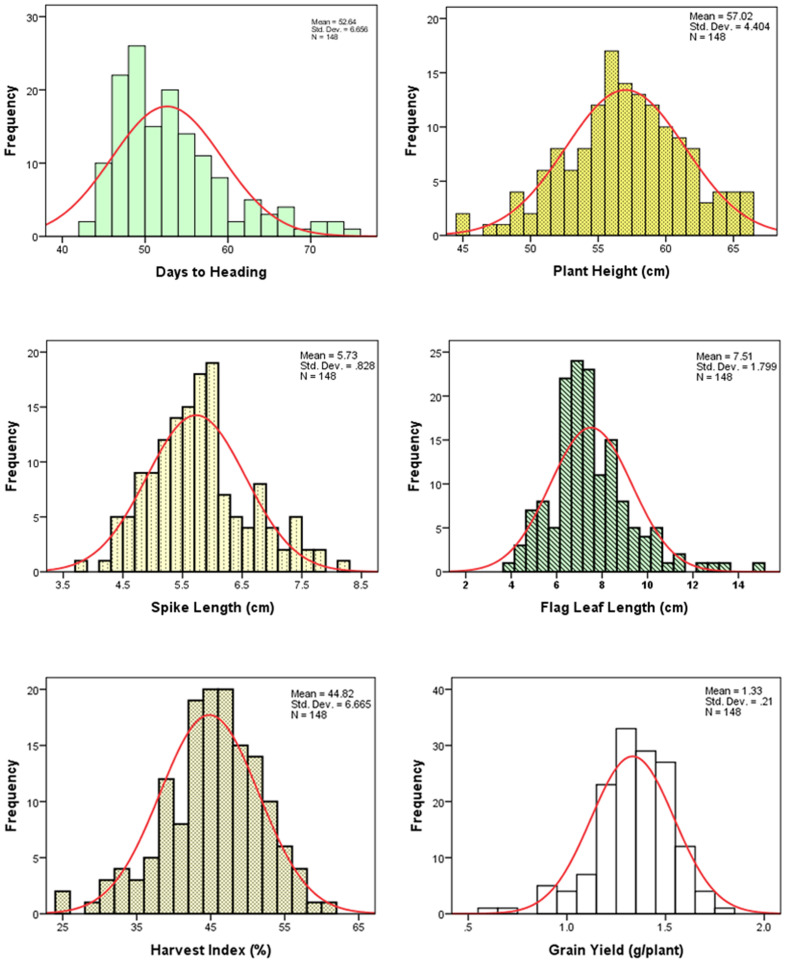
Frequency distribution of the measured traits of 146 doubled haploid (DH) lines derived from the Clipper × Sahara 3771.

The frequency distributions of the measured traits have been shown in **Figure 1**. Analysis of variance revealed a significant difference among the DHs (*P* < 0.01) for the all measured traits (**Table 1**). Similar to DHs, the parental genotypes (Sahara 3771 and Clipper) were significantly different for all traits except HI. A wide range of variation was observed among DHs in the studied traits (**Table 2** and **Figure 1**) and all traits approximately fit normal distributions, indicating the distribution expected for a polygenic and quantitatively inherited trait (Sadeghzadeh et al., 2010; Zhou, 2015). Phenotypic and genotyping coefficient of variation ranged from 8 to 34% and 7 to 32% in the DH population, respectively (**Table 2**). The environmental effects on all morphological traits, GY and its components were small, but the genotypic variance was large for all traits. The large heritability was estimated for SN (93%), DAA (94%), and TKW (97%). Estimates heritability of the traits ranged from 67 to 97%, indicating a high chance of detecting QTL for these traits by using a suitable linkage map.

**Table 1:** Analysis of variance for yield and morphological traits in 146 barley doubled haploid (DH) lines.

SOV	*df*	Mean of square								
		
		Days to awn appearance	Plant height	Spike length	Flag leaf length	Fertile spike	Seed number	Thousands kernel	Harvest index	Grain yield
Block	1	325^ns^	151^ns^	2.8^ns^	12.5^ns^	6.4^ns^	565^ns^	5.6^ns^	231^ns^	0.83^ns^
Genotype	145	8.5^∗∗^	39.7^∗∗^	1.4^∗∗^	5.7^∗∗^	1.21^∗∗^	188^∗∗^	108^∗∗^	90.7^∗∗^	0.09^∗∗^
Error	145	5.30	5.31	0.29	1.90	0.12	12.57	3.29	8.99	0.01
Total	291									

CV%		4	4	9	19	15	10	5	7	9


*“^∗^”, “^∗∗^”, and “ns” are significant at the 0.05, 0.01 levels and non-significant, respectively.*

**Table 2:** The statistics of the 146 lines from DH population based on data from the Clipper × Sahara 3771.

Trait	Parents		Doubled Haploid populations						
		
	Sah	Clip	Range	Mean	LSD (5%)	Genotyping coefficient of variation (GCV)%	Phenotyping coefficient of variation (PCV)%	General heritability (h^2^) %	Genetic advance %
Days to awn appearance	72	49	42–75	52	4.5	12	12	94	24
Plant height (cm)	52	55	45–66	57	4.6	7	8	87	14
Spike length (cm)	7.4	5	3.8–8.1	5.7	1.1	13	14	79	23
Flag leaf length	15	5	3.9–13.4	7.5	2.7	18	23	67	31
Fertile spike	1.0	3.3	1–3.8	2.3	0.7	32	34	90	63
Seed per plant	38	29	15–62	36	7.0	26	27	93	52
Thousand kernel (g)	31	49	24–54	39	3.6	19	19	97	38
Harvest index (%)	45	45	25–60	45	5.9	14	15	90	28
Grain yield (g/plant)	1.1	1.5	0.4–1.8	1.34	0.2	14	16	85	28


A comprehensive molecular map of DH population enabled the identification of QTLs for morphological traits. Twenty-seven QTLs associated with the studied traits were detected on seven chromosomes (**Table 3**). Three QTLs based on mean value of trait were detected for DAA, PLH, FLL, and FSN traits.

**Table 3:** Quantitative trait loci (QTL) detected for morphological traits and yield based on data form ‘Clipper’ × ‘Sahara 3771’ 146 DH population.

Trait	QTL^a^	Ch.	Flanking markers	Size (Mega-base)	Interval QTL position (cM)^b^	Additive effect^c^	Explained variance (%)
Days to awn appearance (DAA)	qDAA-1	4H	ABC305A-HVGLYT5	188.9	114.6–121.9	S 2.7^∗∗∗^	10
	qDAA-2	5H	ABG702-GBMS141	171.6	108.3–120.3	S 4.5^∗∗∗^	33
	qDAA-3	7H	ABC152D-AWBMA12A	86	52.9–64.5	C 1.99^∗∗∗^	11
Plant height (PLH)	qPLH-1	3H	WG178-HVM60	108.5	60–73.9	C 1.62^∗∗∗^	17
	qPLH-2	4H	BCD808C-HVKNOX3	52.9	29.7–44.3	C 1.53^∗∗∗^	17
	qPLH-3	5H	KSUA3A-ABC164	42	21.9–32.9	C 1.36^∗∗∗^	11
Spike length (SL)	qSL-1	1H	ABG74-WG789D	54.3	33.9–56.1	C 0.16^∗∗∗^	3
	qSL-2	3H	WG405-HVGSL8	79.6	43–53.4	C 0.4^∗∗∗^	16
	qSL-3	4H	ANIONT1A-TACMD	143.7	86–96.7	S 0.26^∗∗∗^	9
	qSL-4	5H	ABG702-GBMS141	170.1	99.3–121.9	S 0.23^∗∗∗^	7
	qSL-5	6H	EBMAC787-GBMS180	117.9	60.1–79.5	S 0.23^∗∗∗^	9
	qSL-6	7H	WG789C-SSS1	47.4	20.1–42.9	C 0.20^∗∗∗^	6
Flag leaf length (FLL)	qFLL-1	1H	CDO105-HVUXS2	71.1	57.1–64.9	S 0.66^∗∗∗^	14
	qFLL-2	3H	BMAG6-EBMAC848	79.6	36–51.4	S 0.59^∗∗∗^	13
	qFLL-3	4H	CDO1312-CDO63	159.3	97.7–113.1	S 0.62^∗∗∗^	13
Fertile spike number (FSN)	qFSN-1	2H	CDO474B-VRS1	134.7	96.8–101	C 0.52^∗∗∗^	46
	qFSN-2	4H	ANIONT1A-TACMD	146.9	87–102.2	C 0.18^∗∗∗^	5
	qFSN-3	6H	PSR167-TAM10	102.4	56.2–74.8	C 0.28^∗∗∗^	12
Seed per plant (SN)	qSN-1	2H	VRS1-KSUF15	136.3	99.8–105	S 8.15^∗∗∗^	67
Thousand kernel weight (TKW)	qTKW-1	2H	CDO474B-VRS1	133.4	96.8–101	C 6^∗∗∗^	69
	qTKW-2	6H	PSR167-TAM10	105.6	50.2–76.5	C 1.3^∗∗∗^	4
Harvest index (HI)	qHI-1	1H	PSR158-CDO105	67.6	45.2–78.8	C 1.74^∗∗∗^	6
	qHI-2	2H	CDO474B-VRS1	130.7	93.8–101	S 4.42^∗∗∗^	38
Grain yield (GY)	qGY-1	1H	BCD454-BMAC32	86.3	61.5–91	C 0.05^∗∗∗^	9
	qGY-2	2H	VRS1-KSUF15	137.6	90.8–112.3	S 0.1^∗∗∗^	14
	qGY-3	4H	CDO358-AWBMA29	80.1	46.9–56.3	C 0.06^∗∗∗^	3
	qGY-4	6H	PSR167-TMA10	99.2	56.2–69.4	C 0.09^∗∗∗^	12


*^a^QTL were named according to McCouch (2008). ^b^The most likely location QTL by composite interval mapping (CI). ^c^‘S’ represents inheritance of allele from ‘Sahara 3771’ and ‘C’ from Clipper parents. “^∗∗∗^” Indicates marker effect is statistically different from zero at *P* < 0.001.*

Three QTLs was associated with DAA trait on chromosome 4HS, 5HL, and 7HL and could totally explain 44% phenotyping variation for this trait (**Table 3**). The more effective QTL (qDAA-2) was flanked by RFLP marker ABG702 and SSR marker GBMS141 on chromosome 5HL. The chromosomal region close to marker GBMS141 was reported for controlling time to anthesis (Hussain et al., 2016). DAA was increased in DHs when alleles from Sahara 3771 were present at the identified QTLs. Sahara 3771 reached DAA later than Clipper (23 ± 4.5 days after sowing).

For PLH, majority of DHs were taller than the parents and ranged from 45 to 66 centimeter (**Table 2** and **Figure 1**). Three QTL for PLH were identified on chromosomes 3, 4, and 5 that could explain 45% of total variation for this trait (**Table 3**). Alleles that increased PLH at all three loci were derived from the parent Clipper that was 3 centimeters taller than Sahara 3771.

For SL, a significant variation was observed in DHs (ranged from 3.8 to 8.1 cm), where SL for Clipper and Sahara 3771 were 7.4 and 5 centimeters, respectively. Six QTLs for SL were distributed on all barley chromosomes except 2H (**Table 3**). Half of the alleles increasing SL at the loci on 1H, 3H, and 7H were contributed by Clipper, and explaining 25% of the phenotypic variance. The second QTL (qSL-2) flanked RFLP markers WG405 and HvGsl8 on 3HL could explain 16% of the phenotypic variation. Although, the correlation between PLH and SL was significant and positive (*r* = 0.37^∗∗^), but there was no common QTL between these traits (**Table 4**).

**Table 4:** Phenotypic associations among the measured traits in DH lines based on Pearson correlation coefficient (*r*).

Trait	Days to awn appearance	Plant height	Spike length	Flag leaf length	Fertile spike	Seed number	6/2-row type	Thousand kernel weight	Harvest index
Plant height	-0.12								
Spike length	0.30**	0.37**							
Flag leaf length	0.36**	-0.12	0.02						
Fertile spike	-0.32**	-0.10	-0.38**	-0.27**					
Seed number	-0.16	-0.04	0.03	-0.30**	-0.33**				
6/2-row type	0.08	-0.09	0.09	-0.04	-0.69**	0.81**			
Thousand kernel weight	-0.11	0.13	-0.06	0.07	0.58**	-0.78**	-0.83**		
Harvest index	-0.35**	0.05	0.06	-0.35**	-0.24**	0.79**	0.57**	-0.40**	
Grain yield	-0.41**	0.11	0.00	-0.41**	0.13	0.69**	0.34**	-0.12**	0.82**


*“^∗∗^” Correlation is significant at the 0.01 level (2-detail).*

Three QTLs underlying FLL were found on 1H, 3H, and 4H. The QTL, qFLL-1 nearby RFLP marker CDO105 and SSR marker HVUXS2 accounted for 14% phenotypic variation. The qFLL-2 mapped on 3H accounted for 13% phenotypic variation. The additional regions, qFLL-3 were also identified to be associated with FLL trait on 4H flanked by CDO1312 and CDO63 markers (**Table 3**). All three alleles increasing FLL were from Sahara 3771 that had 10 centimeters larger flag leaf compared with Clipper.

Three QTLs for FSN were mapped on 2H, 4H, and 6H (**Table 3**), explaining 63% of the phenotypic variance. All alleles increasing FSN were contributed by Clipper that had threefold more FSN than that Sahara 3771. Most of variance (46%) in SN was controlled by qFSN-1 located on 2HL flanked by CDO474B-VRS1, and might be utilized in MAS in breeding for higher FSN per plant.

A significant differences was observed among the DHs for SN per plant (ranged from 15 to 62 with an average of 36 seeds per plant). Sahara 3771 (6-row barley) had nine more seeds per plant compared with Clipper (2-row barley). This variation in seed number is mainly due to high seed number of 6-row barley genotypes as compared to 2-row genotypes. In QTL analysis, one region was found to be associated with SN on 2H (VRS1-KSUF15), accounting for 67% of the total variation in SN trait.

Thousands kernel weight of DHs ranged from 24 to 54 g, which shows significant variation among the DHs for seed size. TKW of Clipper and Sahara 3771 were 49 and 31 g, respectively. The variation in distribution of seed size was mainly attributed to differences in spike row-type, with the 2-row plants (cv. Clipper-like) having significantly greater seed weight than the 6-row plants (Sahara 3771-like). QTLs associated with TKW were located on two different chromosomes (2H and 6H) that were contributed by Clipper alleles. In fact, most of TKW phenotypic variation (69%) was controlled by qTKW-1 flanking by CDO474B-VRS-1 markers located on 2HL (**Table 3**).

The largest variation was found for HI among DHs (ranged from 25 to 60), where the both parents were similar for this trait (44.6 and 44.7% for Sahara 3771 and Clipper, respectively). Beneficial loci were donated from both parents, providing a good example of transgressive segregation for HI. Two QTLs were identified for HI on 1H and 2H, explaining 44% of variation in HI. QTL on chromosome 2H was between CDO474B and VRS1 markers, and accounted for 38% of HI phenotypic variation. This QTL was also strongly associated with TKW (**Table 3**).

**FIGURE 2:**
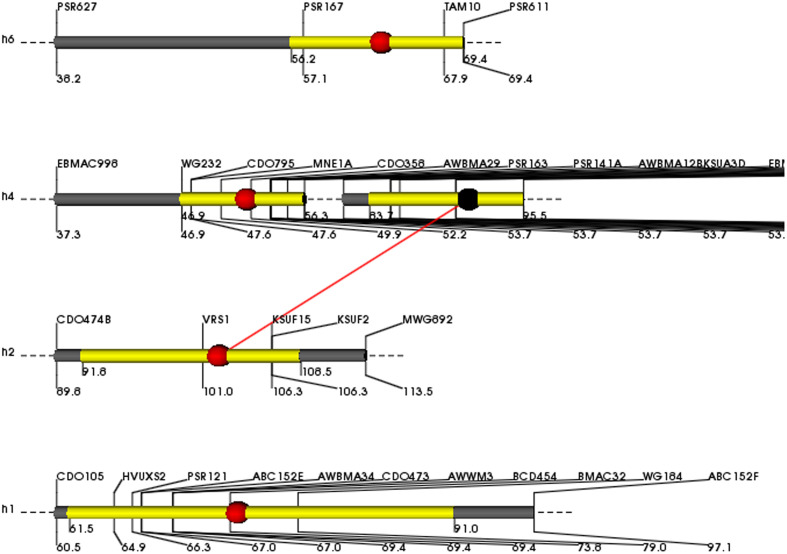
Map positions of grain yield QTLs on 1H, 2H, 4H, and 6H chromosomes expressed in the DH lines. The molecular markers and genetic distance (cM) are identified on the top and beneath side of the chromosome, respectively. The yellow areas show the support intervals of the QTL positions. The red dots on the brighter thick bars indicate the peak positions of the QTLs. Red bar shows epistatic effect between two chromosome regions.

Grain yield was 50% higher in Clipper than Sahara 3771 and ranged from 0.4 to 1.8 g/plant in DHs (**Table 2** and **Figure 2**). Four QTLs associated with GY were detected on 1H, 2H, 4H, and 6H, controlling 38% of total variation of GY (**Table 3** and **Figure 2**). Of them, one QTL (qGY-1) was detected on chromosome 1H, and accounted for 9% of phenotypic variation. The QTL located in interval VRS1-KSUF15 on 2HL explained 14% of yield variation. This QTL was also tightly associated with SN per plant where there was positively significant correlation between GY and SN (*r* = 0.69^∗∗^).

## Discussion

Quantitative trait loci mapping is an efficient method to analyze genetically complex traits and genotype-phenotype mapping (Chengsong et al., 2008). In barley, the correlation and QTLs of agronomic traits affecting GY in DHs derived from Clipper and Sahara 3771 had not been systematically analyzed. Desirable genetic variations for the measured traits in DHs was helpful in identification of the QTLs associated with the traits (**Tables 2**, **3**). New methods in QTL mapping and more available sets of phenotypic data helped in detecting more and novel QTLs for the agronomic and yield component traits.

The DH lines on average were higher in PLH, FSN, and SN than the mean of the two parents, indicating the presence of additive × additive epistasis (Jui et al., 1997). There were significant correlations between DAA, FLL, SN, 6/2-row type, TKW, and HI with GY, but it is not known whether these correlations were due to linkages among QTLs or pleiotropy, or both. General heritability of most traits was above 85%, indicating that screening for these traits would be effective (**Table 2**). SN per plant was positively correlated with GY, showing that SN may be a worthy screening criterion for better GY.

Twenty-seven QTLs were detected on all barley chromosomes. The number of significant QTL(s) per trait ranged from one to six (**Table 3**). In some traits with two or more significant QTLs detected, it can be inferred that both parents carry genes with alleles that increase or decrease phenotypic values (Xiao et al., 1996). Three overlapping QTLs of FS, TKW, and HI were identified on long arm of chromosome 2H flanking between CDO474B and VRS1 markers, explaining 46, 69, and 38% of phenotypic variations for these traits, respectively. Alleles at this locus from higher fertile spike Clipper promoted greater TKW, where there is strong correlation between fertile spike and TKW (*r* = 0.58^∗∗^). Marker VRS-1 controls 6/2 rowed-type in barley spike and confirms that 2-rowed plants have more fertile spike in comparison with 6-rowed plants (Komatsuda et al., 1999; Tanno et al., 2002). The existence of significantly negative correlation between 6-rowed spike and fertile spike (*r* = -0.69^∗∗^) supports the QTL analysis (**Table 4**). Meanwhile, fertile spike with 32% genotypic coefficient variation had the highest value among the measured traits (**Table 2**). As expected, the major contributor to seed size was the 2-row locus (VRS1-KSUF15) on 2HL (originating from Clipper), which had a main controlling effect on seed weight (**Table 3**). This study also showed the existence of negatively large correlation (*r* = -0.83^∗∗^) between 6-row type and TKW (**Table 4**). Similar results were also reported for the role of 2-row locus (Vrs1) and seed size on Clipper × Sahara 3771 originated DH population (Lonergan et al., 2009; Sadeghzadeh et al., 2015).

For GY, co-localization of QTLs and related traits reported in previous studies (Quarrie et al., 2006; Gao et al., 2015). In the current study, QTL flanked by PSR167-TAM10 on 6H controlled FS, TKW, and GY traits (**Table 3**). Moreover, the interval 91–112 cM on long arm of 2H (VRS1-KSUF15) was a pleiotropic locus impacting SN and GY traits. No similar pleiotropic region on 2HL was previously reported. GY showed a significantly positive correlation with SN (*r* = 0.69^∗∗^). QTL contributed to VRS1-KSUF15 in this study had already been reported for GY increase (Hussain et al., 2016). Hence, the identified makers can be used for MAS to improve breeding efficiency. In some studies, this chromosome region (VRS1-KSUF15) was mapped on chromosome 2HL as controlling seed Zn content (Lonergan et al., 2009; Sadeghzadeh et al., 2015). We found that the identified region affected SN per plant, HI and GY. These data confirm efficiency of this chromosomal region to the fine mapping of these traits. Identified QTLs in glasshouse conditions, suggesting their value in marker-assisted selection. However, the assessment in field conditions were not performed in this DH population will be important for obtaining an inclusive view of breeding potential for these traits in barley in natural conditions to extend this research to other main crops.

## Conclusion

The existence of great genotypic variations for the measured traits in Clipper and Sahara 3771 as well as derived DH lines proved the efficiency of this population for dissecting genomic regions influencing agronomic traits and GY components. Under glasshouse growing conditions, 2-row lines (Clipper and Clipper type) had 52% less grain number than 6-row ones (Sahara 3771 and Sahara type); however, 6-row lines (Sahara 3771 and Sahara 3771 type) had 11% more GY than 2-row ones (Clipper and Clipper type). The 6-row lines, however, were associated with low FS and low seed weight. Out of 27 QTLs associated with the interested traits in this study, the identified locus on the long arm of 2H chromosome controlling row type (Vrs1), FSN, SN per plant, TKW, HI, and GY, may facilitate the utilization of molecular markers in MAS in barley breeding programs for improved yield and yield components.

## Author Contributions

UV, the corresponding author of the manuscript. Substantial contributions: design of the work, analysis and interpretation of data for the work and drafting the article. Agreement to be accountable for all aspects of the work in ensuring that questions related to the accuracy or integrity of any part of the work are appropriately investigated and resolved. SHJ design of the work, analysis, drafting the work or revising it critically for important intellectual content, final approval of the version to be published. BS: design of the work; or the acquisition, analysis, final approval of the version to be published. Agreement to be accountable for all aspects of the work in ensuring that questions related to the accuracy or integrity of any part of the work are appropriately investigated and resolved. BAM: design of the work, analysis, drafting the work or revising it critically for important intellectual content, final approval of the version to be published.

## Conflict of Interest Statement

The authors declare that the research was conducted in the absence of any commercial or financial relationships that could be construed as a potential conflict of interest.
